# Exposure and health impacts of outdoor particulate matter in two urban and industrialized area of Tabriz, Iran

**DOI:** 10.1186/2052-336X-12-27

**Published:** 2014-01-10

**Authors:** Akbar Gholampour, Ramin Nabizadeh, Simin Naseri, Masud Yunesian, Hasan Taghipour, Noushin Rastkari, Shahrokh Nazmara, Sasan Faridi, Amir Hossein Mahvi

**Affiliations:** 1 Department of Environmental Health Engineering, School of Public Health, Tehran University of Medical Sciences, Tehran, Iran; 2 Department of Environmental Health Engineering, School of Public Health, Tabriz University of Medical Sciences, Tabriz, Iran; 3 Center for Water Quality Research (CAPR), Institute for Environmental Research (IER), Tehran University of Medical Sciences, Tehran, Iran; 4 Center for Air Pollution Research (CAPR), Institute for Environmental Research (IER), Tehran University of Medical Sciences, Tehran, Iran; 5 Center for Solid Waste Research (CAPR), Institute for Environmental Research, Tehran University of Medical Sciences, Tehran, Iran; 6 National Institute of Health Research, Tehran University of Medical Sciences, Tehran, Iran

**Keywords:** Air pollution, Particulate matter, Health impact assessment, AirQ software

## Abstract

Numerous studies have shown associations between air pollution and health effects on human. The aims of the present study were to provide quantitative data on variation of atmospheric particulate matter (PM) concentration and the impact of PM on the health of people living in Tabriz city. The approach proposed by the World Health Organization (WHO) was applied using the AirQ 2.2.3 software developed by the WHO European Centre for Environment and Health, Bilthoven Division. The concentration of particulate matter were measured at urban and industrial suburban sites in Tabriz, Iran, from September 2012 to June 2013. TSP and PM_10_ samples were collected using high volume samplers. PM_2.5_ and PM_1_ were measured by Haz-Dust EPAM-5000 particulate air monitors. The annual average concentrations of TSP, PM_10_, PM_2.5_, and PM_1_ in the urban site were 142.2 ± 76.3, 85.3 ± 43.9, 39 ± 19.1, and 28.4 ± 14.9 μg/m^3^ (mean ± SD), respectively. Also in industrial suburban, the total average concentrations of TSP, PM_10_, PM_2.5_, and PM_1_ were measured as 178.7 ± 52.7, 109.9 ± 30.2, 40.0 ± 10.9, and 31.4 ± 9.1 μg/m^3^, respectively. The PM_10_/TSP ratio for the whole study period ranged between 0.35-0.91 and 0.32-0.79 in the urban and suburban sites, respectively. Total mortalities associated with TSP, PM_10_ and PM_2.5_ concentrations were 327, 363, and 360, respectively. Furthermore, the cardiovascular mortalities for TSP and PM_10_ were 202 and 227 individual, respectively. According to the attributable respiratory mortalities of 99 and 67 associated respectively with TSP and PM_10_, it is clear that cardiovascular mortality resulted from PM might attributed to total mortality. The maximum 24-hour concentration of PM was observed during winter followed by autumn and the lowest one was during spring.

## Introduction

Industrialization, urbanization and increasing of population are resulting in the pollution of environment [1,2]. Deterioration of urban air quality has become an increasing and widespread concern in both developed and developing communities [3-5]. Among air pollutant, particulate matter (PM) is the pollutant with the most undesired health effects [6]. The most important effects of air pollution on human health have been attributed to the existence of PM at high levels. Not only it does lead to an increased mortality [7,8] and the number of hospital admissions due to different causes; the amount of Quality Adjusted Life Years (QALYs) lost as a consequence of exposure to such particles is estimated by the WHO around 6.4 million per year [9]. Also, it has been shown that heart and respiratory diseases are hazardously affected with environmental particulate matter [7,8,10].

Generally, the fine particulate fraction of total suspended particulates (TSP) (i.e., PM_2.5_) arises from combustion processes. On the other hand, the coarser fraction (i.e., PM_10_) originates from mechanical and re-suspension processes [11]. The deposition of PM at different regions of respiratory system directly depends on particles’ size [12]. While PM_10_ have been associated with respiratory hospital admissions [6,13], PM_2.5_ have been more strongly correlated with both mortality and morbidity [14,15]. Therefore, PM_2.5_ have received more attentions in recent years due to its stronger ability in developing adverse health impacts [8,16,17].

Pope and Dockery summarized evaluations of health effects associated with long- and short-term exposures to ambient PM conducted in recent year; where short-term is referring to 24-hr exposure [8]. They reported that PM_10_ is associated with all-cause mortality, lung cancer, and nonmalignant respiratory mortality for males and coronary heart disease in females [7]. Several studies have focused on the mass concentrations, health impacts, chemical characterization, and source identification of PM and also dust storm in some cities of Iran [18-23]. However, to the best of our knowledge, there are limited published studies, which have dealt with mass concentrations and health impact assessment of PM in Tabriz [24].

**Figure 1:**
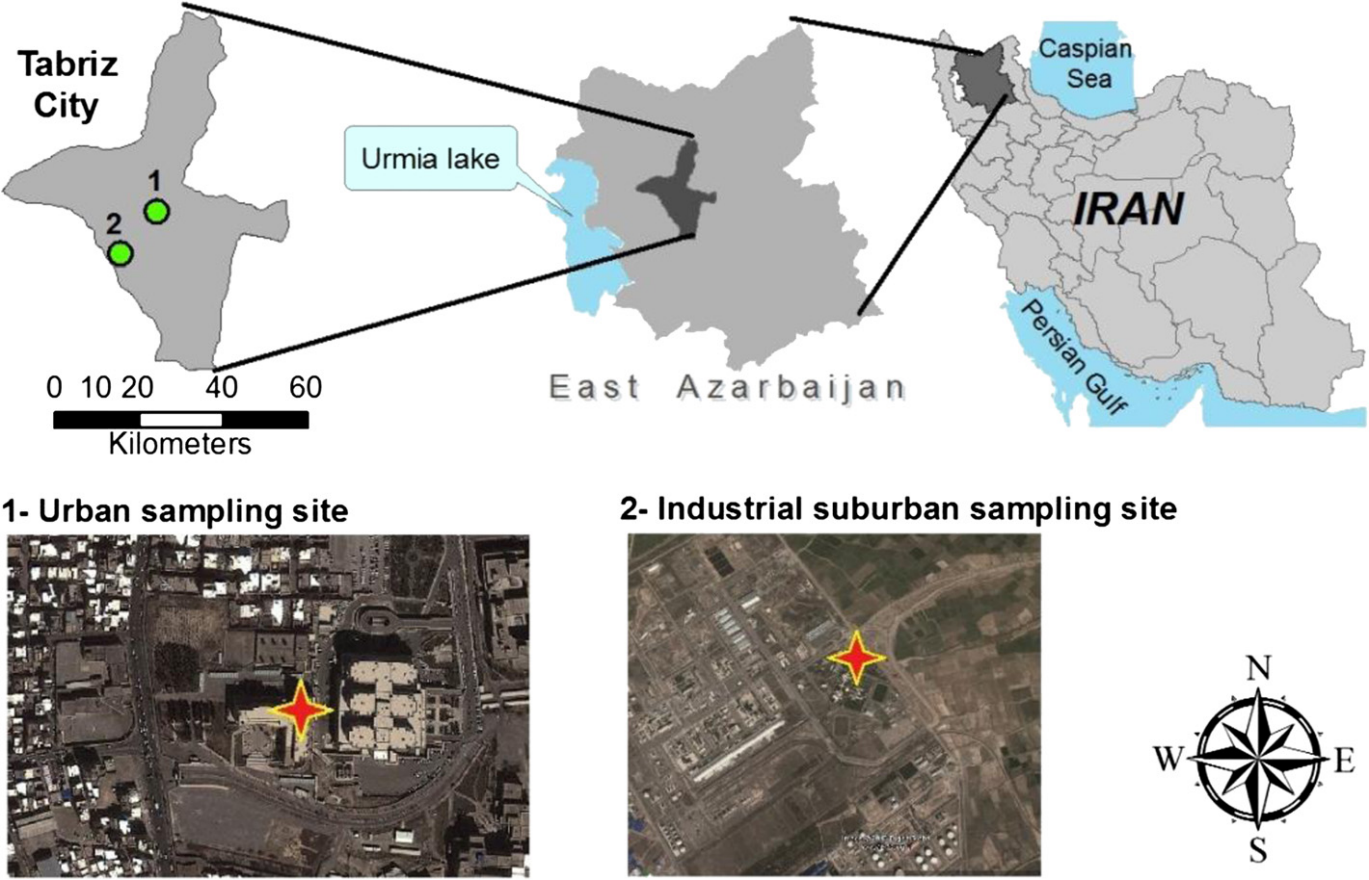
Location of study area and sampling sites.

Tabriz (Figure 1) is the capital city of East Azerbaijan province. It is one of the largest urban areas in Iran with approximately 1.7 million population in 2012 and total surface area of 320 km^2^[25]. There are some light and heavy industries located on the Northwestern, Western, and Southwestern of this city. Industries such as oil refinery, thermal power plant, and petrochemical complex have been located on the Southwestern, while a cement factory has been situated on the Northwestern fringe of the city. In recent years, because of development of industries and also increase in the number of vehicles in urban area, Tabriz has faced with serious air pollution problems, especially in winter season [24]. In addition, the air pollution in Tabriz is mostly under the influence of atmospheric thermal inversion in cold season and moreover recently the Middle East dust storm (originating from Iraq) in the warm season exacerbated the air pollution in this area [24].

The present study was therefore carried out to determine the mass levels of (TSP), PM_10_, PM_2.5_, and PM_1_ along with the seasonal variations of PM in Tabriz. The other aim of this study was to estimate health impacts of TSP, PM_10_ and PM_2.5_ using AirQ software developed by World Health Organization (WHO).

## Materials and methods

### Sampling sites and schedule

Two sites were selected based on their different land use categories: (1) the urban site, located near the center of city in a residential region (38° 3′ 18.08″ N, 46° 19′ 22.77″ E), with the distance about 200 m from major street and 1000 m away from a main freeway [26]. The samplers were operated on the roof of a three-stories building at the height of 15 meters above ground level. Samples were collected on every six day throughout the sampling period [27], from September 2012 to June 2013. (2) An industrial suburban site situated out of the urban border, approximately 1000 m away from a major freeway and 500 m from the main street (38° 4′ 23.98″ N, 46° 9′ 35.55″ E). A petroleum refinery, a small industrial estate, a thermal powerhouse, and some other small industrial plants were located adjacent to the industrial sampling site. The samplers were operated on the height of 3 meters above the ground level. About 3 to 4 samples were collected in every month during the November 2012 to May 2013 [27]. The location of sampling stations is shown in Figure 1.

### PM measurement

Samples of TSP, PM_10_, PM_2.5_, and PM_1_were simultaneously collected during the study period. TSP and PM_10_ samples (n = 80) were collected by two high volume (hi-vol.) samplers (Graseby–Andersen) at flow rates of 1.13–1.41 m^3^/min for 24 h. Both TSP and PM_10_ were collected on a 20.3 cm×25.4 cm Whatman glass micro fiber filters. All filters were maintained at conditions of 40% relative humidity (RH) and 25°C for over 48 h, afterward at the room condition for 2 h and then were weighted before sampling. After collection of PM samples, the filters were treated in the same conditions, which mentioned for preparation of filters and then were weighed using an A&D electronic balance (Model GR-300) with the reading precision of 0.1 mg.

PM_2.5_ and PM_1.0_ (n = 80) were measured by means of two portable HAZ-DUST EPAM-5000 particulate air monitors at the flow rate of 2.5-3 L/min. The Haz-Dust had been calibrated using Arizona Road Dust (ARD) against NIOSH method 0600 for inhaling dust with a ± 10% accuracy in the factory. Also, the span reference was used as a reference to check factory calibration of the EPAM-5000 Monitor.

### Meteorological data

Wind speed, wind direction, ambient air temperature, atmospheric visibility, and RH at sampling stations were obtained from the national climatic data center (NCDC) [28] and East Azerbaijan Meteorological Organization. The obtained data were examined for the missing values and outliers to input in WRPLOT View Freeware 7.0.0 to plot the wind rose and also in Microsoft Excel 2010 to plot the temporal trends for the other parameters.

### Data analysis

Descriptive statistics were used to explain the obtained results. The concentrations of PM were analyzed using Microsoft Excel 2010. Hourly and daily average concentrations were calculated from 30 min data. The mortality and morbidity rates associated with the TSP, PM_10_, and PM_2.5_ concentrations were estimated by AirQ software Ver.2.2.3, which was developed by WHO European Centre for Environment Health [29].

AirQ software was used to estimate the effect of exposure to specific atmospheric pollutants (PM effect in this study) on the health of people living in a certain period and area. The estimation is based on the attributable proportion (AP), defined as the fraction of the health impact in a certain population attributable to the exposure to a given atmospheric pollutant. The formula used for calculation of AP is [30]:

(1) $$\mathrm{AP}=\frac{\sum \left\{\left[\mathrm{RR}\left(c\right)-1\right]\times P\left(c\right)\right\}}{\sum \left[\mathrm{RR}\left(c\right)\times P\left(c\right)\right]}$$

where:

AP is the attributable proportion of the health impact,

RR is the relative risk for a given health impact, in category “C” of exposure which can be obtained from the exposure to response functions derived from epidemiological researches and

P(c) is the amount of population in category “C”.

If the baseline incidence of health impact in the population under study is known, the attributable rate to the exposure can be calculated from following formula:

(2) IE=I×AP

where:

IE is the rate of the health impact attributable to the exposure, and

I is the baseline incidence of health outcome in the population under study.

Finally, knowing the total number of population, the amount of cases attributable to the exposure can be estimated as:

(3) NE=IE×N

where:

NE is the amount of cases attributed to the exposure, and

N is the total number of investigated population.

The RR values used for TSP analysis were derived from the study of Chen *et al.*[31] and Goldberg *et al.*[32], while in the case of PM_10_, this parameter was obtained from a quantitative meta-analysis by Anderson *et al.*[33] and also adopted from the study of Breitner *et al.*[34] and finally a summary estimate in the WHO Air Quality Guidelines for Europe [35] was used for estimating of PM_2.5_ induced health effects.

In this study, exposure was estimated considering the city of Tabriz with a residential population of about 1,700,000 people.

## Results and discussion

**Figure 2:**
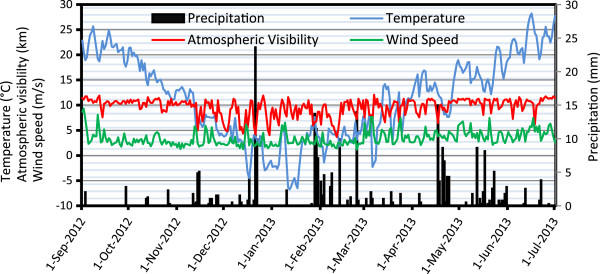
The trend of daily average for ambient temperature, visibility, wind speed, and precipitation in Tabriz.

**Figure 3:**
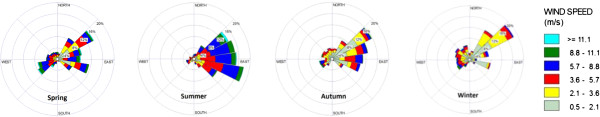
Seasonal wind rose plots during the study period in Tabriz (2012-2013).

The variations of meteorological data including ambient air temperature, atmospheric visibility, precipitation, and wind speed during the study period are shown in Figure 2. Based on the collected data at present study, January was the coldest month with the mean temperature of −3°C, while July was the warmest month with the mean temperature of 38°C. Also the RH varied from 15% to 87%. Seasonal wind rose plots (Figure 3) show that autumn and winter, with the mean wind speeds of 3.12 and 3.08 m/s, respectively, were relatively the calm seasons compared with summer (5.14 m/s) and spring (4.57 m/s). The prevailing wind blew from the Northeast with the speed varied from 0.5 to 11.5 m/s. The annual mean wind speed was 4.01 m/s. Calm wind (0 m/s) frequencies were 5.3%, 1.8%, 11.5%, and 9.35% in spring, summer, autumn and winter, respectively.

In this study, total of 160 samples in the urban and 60 samples in the industrial suburban were collected. Descriptive statistics of mass concentrations of the PM are presented in Table 1. The annual average concentrations of TSP, PM_10_, PM_2.5_, and PM_1_ in the urban sampling site were 142.2 ± 76.3, 85.3 ± 43.9, 39 ± 19.1, and 28.4 ± 14.9 μg/m^3^, respectively. The percentage of days that 24-h mean concentrations of PM_10_ and PM_2.5_ exceeded WHO guideline [36] and national standard level (50 μg/m^3^ for PM_10_ and 25 μg/m^3^ for PM_2.5_) in the cold months were considerably higher than the warm months. Due to weather conditions, decrease of temperature, and higher frequency of calm wind, which led to thermal inversion in cold months (especially October, January, and February), together increase in the use of fuel for heating devices in homes and traffic-related combustion processes resulting in the increase of PMs’ mass concentration. The highest concentration of PM was observed during winter followed by autumn and the lowest concentration was during spring. The obtained results show that the measured maximum diurnal mass concentration of TSP (480.4 μg/m^3^) occurred in the 7th January (in this study) while in the case of PM_10_, maximum diurnal mass concentration (230.0 μg/m^3^) was observed in 14th January. In addition, the maximum daily mass concentration of PM_2.5_ (96.6 μg/m^3^) and PM_1_ (72.2 μg/m^3^) were detected in February.

**Table 1:** **Descriptive statistics for the 24-hour PM mass concentrations (μg/m**^**3**^**) in the urban and suburban sampling sites**

**PM**	**Urban sampling site**							**Suburban sampling site**					
	**Month**	**Average**	**SD**	**Min**	**Max**	**Median**	**% exceed from standard level**	**Average**	**SD**	**Min**	**Max**	**Median**	**% exceed from standard level**
**TSP**	**September**	**143.4**	**60.4**	**67.9**	**227.6**	**146.4**	**--**	**--**	**--**	**--**	**--**	**--**	**--**
	**October**	**209.8**	**71.8**	**113.1**	**313.2**	**209.8**	**--**	**--**	**--**	**--**	**--**	**--**	**--**
	**November**	**70.5**	**65.2**	**26.5**	**183.2**	**37.9**	**--**	**204.9**	**11.4**	**191.8**	**212.8**	**210.00**	**--**
	**December**	**112.0**	**36.1**	**76.4**	**169.9**	**112.0**	**--**	**193.4**	**21.0**	**171.5**	**213.3**	**195.40**	**--**
	**January**	**184.5**	**98.6**	**104.7**	**480.4**	**155.1**	**--**	**219.5**	**61.4**	**154.5**	**276.5**	**227.45**	**--**
	**February**	**178.4**	**55.2**	**101.5**	**255.6**	**178.4**	**--**	**148.1**	**21.1**	**130.4**	**171.4**	**142.50**	**--**
	**March**	**139.5**	**66.5**	**78.6**	**251.7**	**119.9**	**--**	**86.6**	**26.0**	**56.9**	**105.5**	**97.49**	**--**
	**April**	**97.8**	**34.1**	**45.7**	**138.9**	**97.7**	**--**	**183.9**	**10.6**	**173.7**	**194.9**	**183.10**	**--**
	**May**	**93.5**	**10.9**	**75.6**	**105.6**	**95.5**	**--**	**214.3**	**47.3**	**162.6**	**255.3**	**224.99**	**--**
	**June**	**102**	**36.6**	**65.8**	**134.4**	**98.5**	**--**	**--**	**--**	**--**	**--**	**--**	**--**
	**Overall**	**142.2**	**76.3**	**26.5**	**480.4**	**127.5**	**--**	**178.7**	**52.7**	**56.9**	**276.5**	**183.0**	**--**
**PM**_**10**_	**September**	**84.9**	**40.0**	**38.2**	**147.8**	**86.2**	**67**	**--**	**--**	**--**	**--**	**--**	**--**
	**October**	**129.5**	**48.3**	**62.7**	**197.1**	**129.5**	**100**	**--**	**--**	**--**	**--**	**--**	**--**
	**November**	**44.2**	**41.2**	**14.9**	**115.3**	**24.5**	**20**	**142.4**	**13.3**	**131.1**	**157.1**	**139.06**	**100**
	**December**	**84.3**	**21.9**	**65.5**	**121.2**	**79.6**	**100**	**115.1**	**10.9**	**103.3**	**124.6**	**117.42**	**100**
	**January**	**109.7**	**33.0**	**67.4**	**230.0**	**97.4**	**100**	**106.2**	**17.3**	**87.5**	**121.8**	**109.40**	**74**
	**February**	**106.2**	**40.6**	**56.0**	**167.2**	**106.2**	**100**	**111.2**	**21.7**	**98.4**	**136.3**	**98.85**	**100**
	**March**	**95.1**	**46.9**	**50.5**	**174.0**	**80.2**	**80**	**53.1**	**14.4**	**36.7**	**63.8**	**58.80**	**35**
	**April**	**44.2**	**15.8**	**21.3**	**65.9**	**44.2**	**20**	**134.2**	**9.7**	**125.4**	**144.5**	**132.80**	**100**
	**May**	**43.3**	**9.8**	**33.1**	**58.5**	**40.6**	**40**	**106.9**	**19.1**	**85.6**	**122.5**	**112.72**	**100**
	**June**	**47.2**	**18.5**	**30.6**	**71.3**	**49.0**	**25**	**--**	**--**	**--**	**--**	**--**	**--**
	**Overall**	**85.2**	**43.9**	**14.9**	**197.1**	**82.3**	**72**	**109.9**	**30.2**	**36.7**	**157.1**	**108.9**	**--**
**PM**_**2.5**_	**September**	**38.7**	**18.2**	**18.6**	**65.2**	**38.8**	**67**	**--**	**--**	**--**	**--**	**--**	**--**
	**October**	**55.5**	**18.8**	**25.6**	**75.3**	**55.5**	**80**	**--**	**--**	**--**	**--**	**--**	**--**
	**November**	**22.8**	**14.0**	**12.0**	**46.8**	**18.0**	**20**	**43.5**	**6.5**	**36.8**	**49.7**	**44.02**	**100**
	**December**	**41.3**	**22.2**	**15.0**	**76.0**	**41.0**	**80**	**44.9**	**6.4**	**40.5**	**52.3**	**42.00**	**100**
	**January**	**47.7**	**14.0**	**31.7**	**79.0**	**43.2**	**100**	**38.7**	**12.9**	**23.9**	**46.8**	**45.48**	**82**
	**February**	**53.0**	**25.3**	**35.1**	**96.6**	**42.4**	**100**	**47.2**	**9.4**	**38.6**	**57.3**	**45.60**	**100**
	**March**	**40.2**	**12.5**	**23.1**	**56.4**	**41.8**	**80**	**21.6**	**5.6**	**15.6**	**26.7**	**22.42**	**44**
	**April**	**23.8**	**7.6**	**13.5**	**34.7**	**23.3**	**40**	**48.9**	**2.5**	**46.4**	**51.4**	**48.90**	**100**
	**May**	**20.0**	**5.0**	**13.4**	**25.4**	**21.5**	**20**	**35.2**	**3.1**	**32.2**	**38.5**	**34.81**	**87**
	**June**	**24.1**	**6.5**	**15.9**	**38.4**	**24.7**	**30**	**--**	**--**	**--**	**--**	**--**	**--**
	**Overall**	**39.0**	**19.1**	**12.0**	**96.6**	**36.6**	**71**	**40.0**	**10.9**	**15.6**	**57.3**	**41.6**	**--**
**PM**_**1**_	**September**	**24.1**	**13.1**	**10.6**	**45.9**	**23.1**	**--**	**--**	**--**	**--**	**--**	**--**	**--**
	**October**	**30.6**	**9.3**	**15.6**	**40.6**	**30.6**	**--**	**--**	**--**	**--**	**--**	**--**	**--**
	**November**	**14.6**	**6.7**	**8.7**	**25.6**	**14.0**	**--**	**37.2**	**5.9**	**30.8**	**42.3**	**38.64**	**--**
	**December**	**32.7**	**18.3**	**11.3**	**60.0**	**32.7**	**--**	**34.6**	**2.5**	**31.8**	**36.4**	**35.65**	**--**
	**January**	**40.3**	**10.8**	**28.0**	**66.0**	**37.8**	**--**	**30.2**	**10.4**	**18.9**	**39.3**	**32.54**	**--**
	**February**	**40.5**	**18.9**	**23.2**	**72.2**	**35.7**	**--**	**37.9**	**7.9**	**31.6**	**46.8**	**35.38**	**--**
	**March**	**28.3**	**10.8**	**14.9**	**43.1**	**28.3**	**--**	**16.6**	**2.8**	**13.4**	**18.9**	**17.45**	**--**
	**April**	**18.3**	**7.5**	**9.5**	**30.3**	**17.8**	**--**	**37.2**	**5.8**	**31.7**	**43.3**	**36.52**	**--**
	**May**	**13.2**	**3.1**	**9.6**	**17.3**	**13.0**	**--**	**25.7**	**3.3**	**23.5**	**29.6**	**24.15**	**--**
	**June**	**22.3**	**5.5**	**12.6**	**25.8**	**20.5**	**--**	**--**	**--**	**--**	**--**	**--**	**--**
	**Overall**	**28.4**	**14.9**	**8.7**	**72.2**	**28.6**	**--**	**31.4**	**9.1**	**13.4**	**46.8**	**31.5**	**--**

The international convention that has been used defining dust storm intensity is based on ambient air wind speeds and the reduction of atmospheric visibility [37]. In this study, system introduced by Hoffmann *et al.*[35] in combination with atmospheric visibility and ambient air wind speed were used to determine dust storm entrance to the study region and also to classify the intensity of dust storm, i.e. “(sand-) dust storms” with wind speeds 17 m/s and visibility 1000 m, “strong (sand-) dust storms” with wind speeds 20 m/s and visibility 200 m and “serious strong (sand-) dust storms” with wind speeds 25 m/s and visibility 50 m. Based on this classification and continual fine-dust measurements, an extended classification was proposed and extended by two classes based on the content of PM_10_ as follows: “Dusty Air” with an hourly average of PM_10_ higher than 50 μg/m^3^ according to the European limit value for PM_10_ and “Light Dust Storm” with an hourly average of PM_10_ higher than 200 μg/m^3^ (Table 2) [38].

**Table 2:** **Dust storm classification (based on PM**_**10 **_**concentration) **[37]**,**[38]

**Category**	**Visibility (m)**	**Wind speed (m/s)**	**PM**_**10 **_**(μg/m**^**3**^**)**
Dusty air	haze	–	50–200
Light dust storm	<2000	–	200–500
Dust storm	<1000	>17	500–2000
Strong dust storm	<200	>20	2000–5000
Serious strong DS	<50	>25	>5000

Based on obtained meteorological data, the minimum atmospheric visibility was 3700 m that occurred in 11th February. In this day, mean wind speed of ambient air was 1.4 m/s and the concentration of PM_10_ was equal to 167.24 μg/m^3^. It is therefore clear from Tables 1 and 2 and also Figure 2, that during the present study, dust storm did not consider at the studied region.

In order to determination of local dust emission, it was assumed that increased dust concentrations during strong winds under non supra regional dust storm conditions would indicate the emission of local dust into the study area [38]. Based on this definition and with respect to the wind speed (6.1 m/s), atmospheric visibility (4200 m), and also high concentration of TSP and PM_10_ at 7th January, it could be concluded that on this date, local dust occurred.

In the industrial suburban, the overall average of TSP, PM_10_, PM_2.5_, and PM_1_mass concentrations were 178.7 ± 52.7, 109.9 ± 30.2, 40.0 ± 10.9, and 31.4 ± 9.1 μg/m^3^, respectively. Due to the nature of industrial activities in this section and their full time work, the variation of PMs’ concentrations could not be related to the monthly trend and is mainly affected by the type and the quantity of industries’ production along with the duration of those activities. Because of the Iranian New Year holidays in March and hence decline in the manufacturing activity and also increase of rainfall, the values of PM decreased significantly in corresponding days. Among days that the ambient air PM has been measured (based on sampling schedule) and also based on obtained meteorological data only at 7th January local dust occurred.

Table 3 represents the ratio of PMs’ species in the urban and industrial suburban sampling sites. The average of PM_2.5_/PM_10_ ratio in the urban site (0.48) was higher than those for suburban (0.38). It might be due to the higher traffic jams in Tabriz city mixed with the emission from the nearby residential area. The PM_10_/TSP ratio for the whole study period ranged between 0.35 - 0.91 and 0.32 - 0.79 in the urban and suburban sites, respectively.

**Table 3:** **The ratio**^**a **^**of PMs’ species in the urban and industrial suburban site**

	**Urban sampling site**				**Industrial suburban sampling site**			
	**PM**_**10**_**/TSP**	**PM**_**2.5**_**/PM**_**10**_	**PM**_**1**_**/PM**_**10**_	**PM**_**1**_**/PM**_**2.5**_	**PM**_**10**_**/TSP**	**PM**_**2.5**_**/PM**_**10**_	**PM**_**1**_**/PM**_**10**_	**PM**_**1**_**/PM**_**2.5**_
Average^a^	0.60	0.48	0.35	0.72	0.63	0.37	0.29	0.79
Max	0.91	0.82	0.67	0.92	0.79	0.46	0.37	0.88
Min	0.35	0.21	0.16	0.53	0.32	0.23	0.20	0.63

^a^Arithmetic average.

Because of significant differences in particle sources and also other geographical and meteorological conditions, the obtained results from one study cannot be directly compared with the findings of other researches. However, the results of some researches in the other countries were studied and compared with the results of the present study. Other studies found that the PM_10_/TSP ratio in high polluted urban regions was 0.4-0.8, whereas this ratio in rural and suburban sites ranged 0.57 - 0.62 [39-41]. As it can be seen, the range of PM_10_/TSP ratio in our study was wider than other researches.

**Figure 4:**
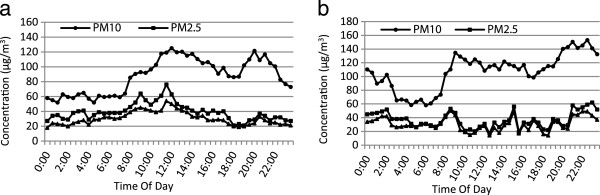
**Average hourly variations of PM**_**10**_**, PM**_**2.5 **_**and PM1 in the urban (a) and industrial suburban (b).**

Figure 4 shows the mean hourly variations of PM_10_, PM_2.5_, and PM_1_ in both sampling sites. As it can be seen in Figure 4, the hourly variations of PM_10_, PM_2.5_, and PM_1_ in urban site have almost an identical behavior and clearly governed by the road traffic pattern. Different patterns were observed for PM_10_, PM_2.5_, and PM_1_ in suburban sampling site compared with the urban site. This difference could be due to the 24-hour working of industries.

High maximum hourly PM_2.5_ and PM_1_ levels at 8 to 12 AM in urban could be due to beginning of daily work and the increased traffic of vehicles. The highest values of PM_10_, PM_2.5_, and PM_1_in suburban site did not show a certain pattern.

**Figure 5:**
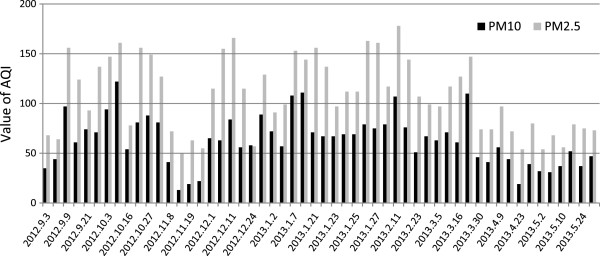
**AQI values in Tabriz for PM**_**10 **_**and PM**_**2.5 **_**during all the days when measurements were made.**

Figure 5 shows the air quality index (AQI) values for PM_10_ and PM_2.5_ concentrations in Tabriz during sampling period. The high PM_10_ concentrations during the October and January produced AQI values over 100. The maximum AQI value calculated for PM_10_ was 122 (for eighth October). These values imply that the air is unhealthy for sensitive groups and may cause the aggravation of cardiovascular and respiratory diseases, increase premature mortality in sensitive groups, and increase hospital admissions for respiratory diseases in the general population. The AQI values for the other days were lower and in the most of sampling days in cold seasons (especially in the winter) were between 50 and 100. These values represent moderate health concern for a very small number of people [42].

For PM_2.5_, the maximum AQI value was 178, and the index values tend to be higher than those for PM_10_. The AQI values for PM_2.5_ generally range from 50 to 150, whereas the AQI values for PM_10_ in most of days were less than 100. These results imply that the corresponding PM_2.5_ AQIs were relatively high and this pollutant poses higher health hazards to the population as compared with PM_10_. As it can be seen (Figure 5), AQI values decrease strongly in spring and result in health effects of PM decline in this season.

These values for PM_2.5_ result from the higher contribution of the fine fraction to the PM_10_ mass. This can be due to high portion of PM_2.5_ results from use of fuel for heating devices in homes and traffic-related combustion processes during cold season in urban.

Based on the measured TSP, PM_10_ and PM_2.5_ concentrations over the study period, some estimated short term health effects of PM are given in Table 4. Relative risk (RR) gives the increase in the probability of the adverse effect associated with a given change in the exposure levels, and comes from time-series studies, where day-to-day changes in air pollutants over long periods were related to the daily mortality, hospital admissions and other public health indicators [11]. Table 4 shows the values of RR used in the present assessment together with total mortality, cardiovascular mortality, respiratory mortality, and some other health effects.

**Table 4:** **Estimated attributable proportion (AP) expressed as percentage and number of excess cases in a year due to short-term exposure to TSP, PM**_**10 **_**and PM**_**2.5**_

	**Relative risk**	**Baseline incidences**	Estimated attributable portion percentage*	Estimated number of excess cases*
	Mean*			
	TSP			
Total mortality	1.003(1.002-1.007)	543.5	3.9(2.6-8.6)	327(221–725)
Cardiovascular mortality	1.002(1–1.006)	497	2.63(0–7.5)	202(0–575)
Respiratory mortality	1.008(1.004-1.018)	66	9.7(5.1-19.5)	99(52–199)
Hospital admissions for respiratory diseases	-----	-----	-----	-----
Hospital admissions for chronic obstructive pulmonary disease	1.0044(1–1.0094)	101.4	5.6(0–11.3)	88(0–176)
Hospital admissions for cerebrovascular disease	-----	-----	-----	-----
	PM_10_			
Total mortality	1.006(1.004-1.008)	543.5	4.3(2.9-5.7)	363(246–478)
Cardiovascular mortality	1.009(1.005-1.013)	231	6.4(3.6-8.9)	227(130–319)
Respiratory mortality	1.013(1.005-1.02)	48.4	8.9(3.6-13)	67(27–98)
Hospital admissions for respiratory diseases	1.008(1.0048-1.0112)	1260	5.7(3.5-7.8)	1107(680–1515)
Hospital admissions for chronic obstructive pulmonary disease	-----	-----	-----	-----
Hospital admissions for cerebrovascular disease	1.009(1.006-1.013)	436	6.3(4.3-8.9)	428(291–601)
	PM_2.5_			
Total mortality	1.015(1.011-1.019)	543.5	4.3(3.2-5.4)	360(267–450)
Cardiovascular mortality	-----	-----	-----	-----
Respiratory mortality	-----	-----	-----	-----
Hospital admissions for respiratory diseases	-----	-----	-----	-----
Hospital admissions for chronic obstructive pulmonary disease	-----	-----	-----	-----
Hospital admissions for cerebrovascular disease	-----	-----	-----	-----

*Mean (lower-upper).

The total mortalities associated with TSP, PM_10_ and PM_2.5_ concentrations are 327, 363 and 360, respectively. Also the cardiovascular mortality for TSP and PM_10_ are 202 and 227, respectively. According to the respiratory mortality associated with the TSP and PM_10_, 99 and 67, respectively, it’s clear that cardiovascular mortality has the main rate in total mortality resulted from PM.

It should be noted that the current study did not include an epidemiological investigation. Therefore, the estimates of health effects were based on the relative risks and the baseline incidences calculated by AirQ2.2.3 software. Since there was not any study to calculate the RR values in Iran, we used the values of RR from the above mentioned studies and also the study of Naddafi *et al.*[6]. Because the characteristics of the target population differed from those of the populations investigated in these epidemiological studies, this approach adds considerable uncertainty to the estimates calculated by the software. However, this information could be highly useful because they provide valuable information about the importance of air pollution and the substantial impacts of PM on the society for policymakers.

## Conclusion

The present study was carried out to determine the mass levels of TSP, PM_10_, PM_2.5_, and PM_1_ and also seasonal variations of water soluble ionic species associated with TSP and PM_10_ particles in urban and industrial suburban sites of Tabriz. The annual average concentrations of TSP, PM_10_, PM_2.5_, and PM_1_ in the industrial suburban were higher than urban sites. In the urban sampling stations, the 24-h mean concentrations of PM_10_ and PM_2.5_, exceeded WHO and national air quality level in 72% and 71% of the time, respectively.

Sometimes local dust occurred in urban and suburban, but dust storm was not observed in this city during the period of present study.

The PM_10_/TSP ratios, for the whole study period, were ranged from 0.35 to 0.91 for urban and from 0.32 to 0.79 for suburban sites, respectively.

In this study we have estimated the impact of PM on human health with AirQ software developed by WHO. Based on the calculated effects, the total mortalities associated with the TSP, PM_10_ and PM_2.5_ concentrations were 327, 363 and 360, respectively. According to the cardiovascular and respiratory mortalities associated with TSP and PM_10_, it might conclude that the cardiovascular mortality has the main role in the total mortality resulted from PM.

## Competing interests

The authors declare that they have no competing interests.

## Authors’ contributions

AG was the main investigator, designed and performed the study and drafted the manuscript. AHM supervised the study. RN, SN, MY, HT, and NR were advisors of the study. SF and ShN helped in the PM analysis. All authors read and approved the final manuscript.
